# PAC1 Receptor Mediates Electroacupuncture-Induced Neuro and Immune Protection During Cisplatin Chemotherapy

**DOI:** 10.3389/fimmu.2021.714244

**Published:** 2021-09-06

**Authors:** Shanshan Li, Jin Huang, Yi Guo, Jiaqi Wang, Shanshan Lu, Bin Wang, Yinan Gong, Siru Qin, Suhong Zhao, Shenjun Wang, Yangyang Liu, Yuxin Fang, Yongming Guo, Zhifang Xu, Luis Ulloa

**Affiliations:** ^1^Research Center of Experimental Acupuncture Science, Tianjin University of Traditional Chinese Medicine, Tianjin, China; ^2^School of Traditional Chinese Medicine, Tianjin University of Traditional Chinese Medicine, Tianjin, China; ^3^National Clinical Research Center for Chinese Medicine Acupuncture and Moxibustion, Tianjin, China; ^4^Tianjin Medical University Cancer Institute and Hospital, National Clinical Research Center for Cancer, Key Laboratory of Cancer Prevention and Therapy, Tianjin’s Clinical Research Center for Cancer, Tianjin, China; ^5^School of Acupuncture & Moxibustion and Tuina, Tianjin University of Traditional Chinese Medicine, Tianjin, China; ^6^Center for Perioperative Organ Protection, Department of Anesthesiology, Duke University, Durham, NC, United States

**Keywords:** neuromodulation, chemotherapy, immunosuppression, hematopoiesis, neurotoxic, electroacupuncture

## Abstract

Platinum-based chemotherapy is an effective treatment used in multiple tumor treatments, but produces severe side effects including neurotoxicity, anemia, and immunosuppression, which limits its anti-tumor efficacy and increases the risk of infections. Electroacupuncture (EA) is often used to ameliorate these side effects, but its mechanism is unknown. Here, we report that EA on ST36 and SP6 prevents cisplatin-induced neurotoxicity and immunosuppression. EA induces neuroprotection, prevents pain-related neurotoxicity, preserves bone marrow (BM) hematopoiesis, and peripheral levels of leukocytes. EA activates sympathetic BM terminals to release pituitary adenylate cyclase activating polypeptide (PACAP). PACAP-receptor PAC1-antagonists abrogate the effects of EA, whereas PAC1-agonists mimic EA, prevent neurotoxicity, immunosuppression, and preserve BM hematopoiesis during cisplatin chemotherapy. Our results indicate that PAC1-agonists may provide therapeutic advantages during chemotherapy to treat patients with advanced neurotoxicity or neuropathies limiting EA efficacy.

## Introduction

Platinum-based chemotherapy, such as cisplatin, carboplatin, and oxaliplatin are widely used in multiple tumors (1–3), but they produce severe side effects including neurotoxicity (4), anemia (5), immunosuppression (6), nephrotoxicity (7), and gastrointestinal toxicity (8). Cisplatin-induced neurotoxicity has been associated with pain neuropathies and deficient neuromodulation contributing to multiple disorders. Cisplatin-induced immunosuppression limits anti-tumor immune responses, treatment efficacy, and increases the risk of infections (9, 10). Thus, chemotherapy is often combined with complementary treatments to prevent immunosuppression, such as EA or treatment with stimulating factors such as colony stimulating factor (CSF) to promote myeloid cell differentiation in the bone marrow (BM) (11). However, CSF is not effective in restoring the proliferation of hematopoietic stem/progenitor cells (HSPCs), induces multiple complications such as bone pain (12), and increases the risk of tumor growth and metastasis by inducing myeloid-derived suppressor cells (13, 14). Thus, there is an unmet clinical need to find safe and effective adjuvant treatments for chemotherapy-induced neurotoxicity and immunosuppression.

Acupuncture is a common complementary and integrative therapy as proved by profuse clinical studies and used by millions of people worldwide (15). The World Health Organization recommends acupuncture to prevent toxicity and leukopenia during radio- and chemotherapy (16). Acupuncture is safe and its effects have been confirmed in multiple clinical trials with different types of tumors including breast (17) and lung cancer (18). Systematic analysis of 31 clinical trials showed that acupuncture alleviated chemotherapy-induced myelosuppression (leukopenia, hemoglobin, and platelet reduction) and preserved immune responses including IL-2 production and lymphocyte counts in lung cancer patients during chemotherapy (19). A pilot, randomized, sham-controlled clinical trial also showed that acupuncture reduced chemotherapy-induced leukopenia in patients with ovarian cancer (20). However, the use of acupuncture is still debated because its inefficacy in some patients. Despite its clinical implications, the mechanism of acupuncture to treat chemotherapy-induced leukopenia is still unknown, and thus its efficacy in many patients but not in others with similar symptoms.

Although the mechanism of acupuncture is unknown, multiple studies reported the critical role of the sympathetic nervous system to modulate BM hematopoiesis. Hematopoietic stem (HSCs) and HSPCs reside in specific BM niches with a complex cellular and molecular environment including mesenchymal stem cells (21), osteoblasts (22), endothelial cells (23), and sympathetic projections (24, 25). Among these, the sympathetic projections are the most critical factors orchestrating BM cell proliferation, differentiation, and egress (24–26). Sympathetic terminals produce multiple factors orchestrating different cell types depending on the physiologic needs. Neurogenic factors produced by these terminals induce different factors such as catecholamines (dopamine and epinephrine) can activate HSCs proliferation and differentiation. The sympathetic system also modulates BM hematopoiesis indirectly by evoking multiple cells to produce stimulating factors, such as granulocyte-colony stimulating factor, which enhances hematopoietic cell proliferation and migration (24). Conversely, sympathetic signals can also inhibit CXCL12 production in mesenchymal stem cells and osteoblasts to induce BM egress of HSCs (27–29). Thus, sympathetic innervations induce complex signals to orchestrate the proliferation, differentiation, and egress of multiple cell types at different levels depending on the physiological needs (27–29). This complexity has made it difficult to design alternative treatments for patients with limited response to acupuncture.

Pituitary adenylate cyclase activating polypeptide (PACAP) is a multifunctional neuropeptide of the glucagon-secretin-vasoactive intestinal peptide (VIP) family, with 67% similarity to VIP (30). There are two isoforms of PACAP: PACAP27 and PACAP38, with the latter being dominant in mammalian tissues in most physiological and pathological conditions (31–33). However, several studies found PACAP levels of different tissue samples are altered under pathological conditions, with lower PACAP immunoreactivity in different human samples of primary small cell lung cancer, colon, and kidney cancers as compared to healthy tissues, while higher PACAP27 immunoreactivity was found in prostatic cancers as compared to benign prostatic hyperplasia (32, 33). PACAP binds to three G-protein coupled receptors, a higher affinity PACAP-specific receptor (PAC1), and two VIP/PACAP receptors (VPAC1 and VPAC2) with similar affinity for VIP and PACAP (34). PACAP has been found to be involved in neuroprotection, prevents apoptosis (35, 36), promotes cell proliferation (37), neurogenesis and axonal regeneration in the central and peripheral nervous systems (38, 39), and modulates immune and inflammatory responses (40, 41). We previously reported that PACAP is secreted by sympathetic nerve endings projected into the BM, and can modulate HSPCs proliferation *via* PAC1 signaling (42).

Multiple studies have shown that cisplatin chemotherapy causes neurotoxicity and multiple neuropathies (43, 44). We reasoned that this neurotoxicity can prevent sympathetic neuromodulation of BM hematopoiesis and thereby induce immunosuppression and leukopenia. In line with our hypothesis, BM hematopoiesis is prevented by neurotoxic agents such as 4-methylcatechol or glial-derived neurotrophic factor and chemotherapy-induced BM nerve injury impairs hematopoietic regeneration (45). Thus, we reasoned that electroacupuncture (EA) may activate BM sympathetic fibers, and protect them from chemotherapy-induced neurotoxicity to preserve hematopoiesis during chemotherapy. Here, we analyze whether EA induces sympathetic neuroprotection and preserves BM hematopoiesis in normal and cancer mice with Lewis lung carcinoma (LLC) cells. We also identify the neurogenic factor that mediates the protective effects of EA during chemotherapy.

## Materials and Methods

### Animals

All experimental procedures were performed in accordance with the Tianjin University of Traditional Chinese Medicine guidelines for the care and use of laboratory animals, and approved by the Animal Care and Use Committee of Tianjin University of Traditional Chinese Medicine (Permit Number: TCM-LAEC2019057). Male Balb/c (8 weeks old, *n*=180) and C57/BL6 (6 weeks old, *n*=50) mice weighting 18-24 g were purchased from the experimental animal center of Beijing Wei Tong Li Hua Experimental Animal Technology Co., Ltd. (Beijing, China). License number: SCXK (Beijing) 2016-0006. All mice were maintained under a 12/12-hour light/dark cycle at 24-26°C in cages at a controlled humidity of 40-50%, and allowed free access to food and water. All mice were anesthetized with 4% isoflurane with oxygen as the carrier (Shenzhen RWD Life Technology Co., Ltd. China) before sacrificing for sample collection.

### Materials and Reagents

Cisplatin (Jiangsu haosen pharmaceutical group Co., Ltd., China) was administered at 3-5 mg/kg in 0.9% sodium chloride solution intraperitoneal (i.p.), twice per week for two weeks. Control mice were treated with an equal amount of saline solution. The role of PACAP was analyzed by using PACAP6-38, a PAC1 antagonist at different concentrations (Low dose:10 μg/kg, High dose:100 μg/kg, i.p., Selleck Chemicals, Houston, USA), and PACAP1-38, PAC1 agonist (Low:10 μg/kg, High:50 μg/kg, i.p., Selleck Chemicals, Houston, USA) (46).

### Establishment of LLC-Bearing Mice Model

LLC cells were cultured with Dulbecco’s modified Eagle’s medium (Gibco, Waltham, MA, USA) supplemented with 10% fetal bovine serum (FBS), 100 μg/ml penicillin, and 0.1 mg/ml streptomycin, and were maintained in a humidified chamber at 37°C in a 5% CO_2_ atmosphere. One week after the C57/BL6 mice are acclimated and injected 1×10^5^ LLC cells in 0.1 ml phosphate-buffered saline (PBS) buffer subcutaneously into the right groin (47). Tumor dimensions were measured by digital calipers at days 7, 10, 14, 17, and 21, and the tumor volume (mm^3^) was calculated as (length × width^2^)/2 (48).

### Electroacupuncture Treatment

EA treatment was initiated on the same day that the mice received cisplatin. Mice were restrained using the soft cloth fixation method, the skin around the bilateral acupoint ST36 (49, 50) (*Zusanli* acupoint, located 2.0 mm lateral to the anterior tubercle of the tibia in the anterior tibial muscle and 4.0 mm distal to the knee joint lower point) and SP6 (51) (*Sanyinjiao* acupoint, located 2.0 mm proximal to the upper border of the medial malleolus, between the posterior border of the tibia and the anterior border of the Achilles tendon) were disinfected with alcohol swabs. The acupuncture needles (diameter=0.25 mm, length=13 mm, Huatuo Brand, Suzhou Medical Appliance Factory, Jiangsu, China) were inserted in bilateral ST36 and SP6 acupoints, with 3.0 and 2.0 mm depth, respectively. Then, the needles were connected to the SDZ-V EA device (Huatuo Brand, Suzhou Medical Appliance Factory, Jiangsu, China) with the dilatational wave at 5/25 Hz and 0.76 mA stimulation for 15 min. Experimental mice received EA three times per week for two weeks, and control mice received the same treatment without EA stimulation.

### Blood Examination

Peripheral blood was collected in polypropylene tubes with ethylenediaminetetraacetic acid (Beijing Nobleryder technology co. Ltd. China) from the orbital sinus of mice anesthetized with isoflurane. Hematological parameters including leukocyte and lymphocyte counts were measured by an automated hematology analyzer (MEK-7222K, Nihon Kohden, Japan).

### Flow Cytometry Assay

#### Hemocyte Panel

200 μL of blood was collected from each sample and incubated with cell membrane markers including LY-6G-PE, LY-6C-APC, CD3-PE-Cy7 and CD19-FITC (Biolegend, San Diego, California, USA) for 20 min at room temperature protected from light. Then, lysing buffer (BD Bioscience, Franklin Lakes, New Jersey, USA) was used to remove red blood cells. Samples were washed before resuspension in 0.5 ml PBS containing 2% FBS. The acquisition was conducted on an Attune™ NxT Acoustic Focusing Cytometer (Thermo Fisher Scientific, Waltham, MA, USA), and the concentration of target population (events/μL) were analyzed.

#### HSPCs Subpopulation Panel

Mice tibias were harvested, the epiphyses of the bones were cut and immersed in 15 ml conical tubes with 1.0 ml PBS. Total BM cells were collected by centrifugation at 3,000×rpm for 10 min, and red blood cells were removed with lysis buffer. For HSPC subsets detection, 10^6^ cells were stained with FITC-conjugated anti-Lin, PE-Cy7-conjugated anti-Sca-1, APC-conjugated anti-CD34, Brilliant Violet 421™-conjugated anti-CD16/32, PE-conjugated anti-CD127 (IL-7R) or Brilliant Violet 510™-conjugated anti-CD127, APC-Cy7-conjugated anti-CD117 (c-Kit), PerCP-Cy5.5-conjugated anti-CD90.1 (Thy1.1), PE-Cy5-conjugated anti-CD135 (Flk2) or PE-conjugated anti-CD135 (Biolegend, San Diego, California, USA) for 20 min at room temperature protected from light. Samples were then washed again before resuspension in 0.5 ml PBS containing 2% FBS. Acquisition was conducted on an Attune™ NxT Acoustic Focusing Cytometer (Thermo Fisher Scientific, Waltham, MA, USA). All the data were analyzed as following: Positive cells events (%) = (the events in target gate/the total cell) × 100.

#### Cell Cycle Panel

Cell cycle was determined by nuclear staining with propidium iodide (PI) of BM cells. Briefly, suspensions of single cells were fixed in 75% ethanol at -20°C overnight. Samples of cells were incubated with 0.5 ml PI (TxCyclePI/RNAse, BD Bioscience, Franklin Lakes, New Jersey, USA) at 4°C for 15 min. Acquisition was conducted the same as the above panel, and analyzed by ModFit 3.1 software (Verity Software House, Topsham, ME).

### Hematoxylin & Eosin (H&E) Staining

Tibial bone was collected and fixed as described (42). The OCT-embedded bone samples were sliced to a thickness of 5.0 μm with a Lecia frozen slicer (1950) and were stained with H&E. The histological sections were observed and photographed under a light microscope (NIKON Eclipse Ci-L, Japan), the section within each group (*n*=4, each sample has two tissue slices) of randomly selected perspective in three pictures. Then BM hematopoietic cellularity was analyzed by Image-Pro Plus 6.0 software (Media Cybernetics, Inc., Rockville, MD, USA) and calculated as follows, BM hematopoietic cellularity (%) = [1 - (white area pixels/total area pixels)] × 100% (52). BM cell density was measured using StrataQuest v7.0.176 software (TissueGnostics, Vienna, Austria). Total cells were identified based on hematoxylin staining. The number and density of cells were counted by the software after excluded cell debris, and BM cell density=total cells counts/total areas (mm^2^).

### Gene Chip (GCT) and Data Analysis

BM sample extraction (*n=*6) was performed as described above in flow cytometry assay. RNAs were extracted purified with a standard Affymetrix protocol according to Shanghai Biotechnology Corporation (Shanghai, China), and equal amount of RNA from each sample was pooled (*n*=1) in the same group and tested on a microarray. The raw chip data are accessible from the BioProject ID PRJNA 687726 in the public database of the NCBI BioProject (https://www.ncbi.nlm.nih.gov/bioproject/PRJNA687726). Briefly, total RNA was isolated and RNA integrity number (RIN) value to inspect RNA integration was checked (53). Only RNA with RIN value greater than 7.0 and a 28S/18S ratio greater than 0.7 were used for microarray analyses. The gene chip results were scanned by Gene Chip Scanner 3000 (Cat#00-00213, Affymetrix, Santa Clara, CA, US) and analyzed by Command Console Software 4.0 (Affymetrix, Santa Clara, CA, US), the qualified data were normalized at the gene and exon levels, respectively by the Expression Console software (Affymetrix, Santa Clara, CA, US) (53), and the normalized signal value was the signal value calculated by Log_2_. Then the differentially expressed genes (DEGs) were screened by threshold method, and the genes with a fold change (FC) > 2 were considered as DEGs as shown in **Figure 3A** of scatter plot. Also, the Kyoto Encyclopedia of Genes and Genomes (KEGG) pathways obtained in the drawing by DEGs through website in http://enrich.shbio.com/. The KEGG obtained were sorted in descending order of size according to the value of the enriching factor and considering the top 30 pathways. The protein-protein interaction (PPI) network is based on the above analysis results of DEGs of different groups (Cis *vs* Veh, EA *vs* Cis) were further analyzed by STRING.

### Reverse Transcription-Quantitative and Polymerase Chain Reaction (RT-qPCR)

RNA samples from each group returned by the company were verified by RT-qPCR. The RNA concentration was measured by Agilent Bioanalyzer 2100 (Agilent Technologies, Santa Clara, CA, US) and total RNA was used for reverse transcription with the PrimeScript RT reagent kit (Takara Bio, Inc., Otsu, Japan) following the manufacturer’s protocol.

The cDNA was amplified by SYBR™ Select Master Mix (Applied Biosystem, Thermo Fisher Scientific, Inc), and the RT-qPCR procedure according to the manufacturer’s protocol. Applied ABI Quant Studio 3 - Time PCR System (Applied Biosystems; Thermo Fisher Scientific, Inc.) was used to perform RT-qPCR under the following conditions: 95°C for 30 sec, followed by 40 cycles of 95°C for 5 sec and 60°C for 30 sec, and finally the melt curve stage (95°C for 15 sec, 60°C for 1 min and 95°C for 15 sec). The associated primers were synthesized by Suzhou GENEWIZ Biological Technology Co. Ltd, which were listed in **Additional File1: Table S1**. Relative gene expression was calculated using the double-standard curve method.

### Immunofluorescence Staining

The bone fixation method was consistent with HE staining. The bone slice thickness of 8.0 μm was rinsed with 0.05% PBST and Proteinase K (BOSTER, WuhFan, China) incubation antigen-repaired for 15 min at room temperature. The following experimental method of immunofluorescence staining was referred to our previous protocol (42). Briefly, the sections were incubated with the primary antibodies rabbit anti-Th (1:50, BOSTER, Wuhan, China) overnight at 4°C. After a 0.05% PBST rinsed, the sections were incubated with Alexa Fluor 594-labeled anti-rabbit IgG (1:400, Abcam, Cambridge, UK) as secondary antibodies for 60 min at room temperature. The sections were observed and photographed under a fluorescence microscope (NIKON Eclipse Ci-L, Japan). Th^+^ immunofluorescence staining analyzed the mean number of nerve fibers in five fields randomly was quantified and plotted as per mm^2^ (45).

### ELISA

BM samples were crushed while frozen and then suspended in cell lysis buffer (Solarbio life sciences, Beijing, China) with protease inhibitor cocktail (1%; Solarbio life sciences, Beijing, China), standing for 30 min at 4°C. Next, samples were centrifuged at 12,000 g for 10 min at 4°C for protein extraction and the clear supernatant extracts were stored at -80°C. PACAP levels including PACAP27 and PACAP38 were measured by using a sandwich enzyme immunoassay special for mouse (Product No. SEB347Mu, Cloud Clone Corp, Wuhan, China) according to the manufacturers’ instructions.

### Heated Pad Assay

Latency time response of mice to thermal nociception was analyzed with hot-plate tests performed at day 0, 3, 7, 10, 14 (54). The hot-plate temperature was set at 55 ± 0.2˚C. Mice were individually placed on the top of the heated surface and the time of the first episode of nociception (jumping or paw licking) was measured, and the cut-off time was 30 s. The heated surface was cleaned up completely by ethanol in two tests and the temperature was allowed to stabilize.

### Statistical Analysis

Results are presented as the mean ± standard error of mean (SEM). When the data were normally distributed, the results were analyzed by analysis of variance (ANOVA) for independent samples compared differences between two groups. Comparison of weight, latency, tumor volumes were assessed two-way repeated-measures ANOVA, other indicators were assessed one-way ANOVA. LSD test was used if the data meet test of homogeneity of variances, if not, Dunnett’s T3 test was used. For non-normal distributions, a nonparametric test with Kruskal Wallis was performed with SPSS 23.0. *P* < 0.05 was considered statistically significant. GraphPad Prism software (GraphPad, San Diego, CA, USA) was used for mapping.

## Results

### Prevention of Cisplatin-Induced Leukopenia and Normal Hematopoiesis Preservation by Electroacupuncture

**Figure 1:**
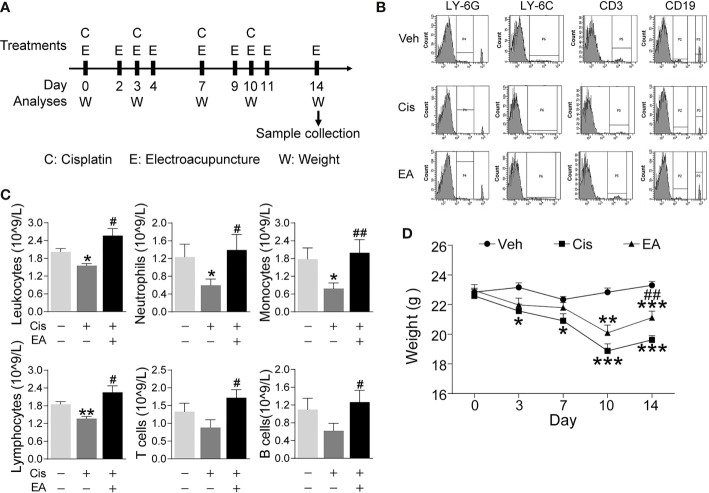
Electroacupuncture prevented cisplatin-induced leukopenia. **(A)** Experimental flowchart depicting the time of the treatments of Cisplatin (C), electroacupuncture (E), and the analyses of body weight (W) and sample collection. **(B)** Representative peripheral blood flow cytometry analyses of neutrophils (LY6G^+^), monocytes (LY6C^+^), T (CD3^+^), and B (CD19^+^) lymphocytes and **(C)** Blood counts of specific subpopulation of leukocytes of mice with control (Veh), cisplatin alone (Cis; 3 mg/kg), or with electroacupuncture (EA) treatment (leukocytes, lymphocytes: *n*=6 per group; neutrophils, monocytes, T and B lymphocytes: Veh, *n*=6; Cis, *n*=6; EA, *n*=7). **(D)** Mice body weight curves treatment at day 0, 3, 7, 10, 14 (*n*=6 per group), *P* values were calculated using two-way repeated-measures ANOVA. Data are mean ± SEM, ^*^ *P* < 0.05, ^**^ *P* < 0.01, ^***^ *P* < 0.001 *vs* Veh; ^#^ *P* < 0.05, ^##^ *P* < 0.01 *vs* Cis.

First, we analyzed whether EA prevents cisplatin-induced leukopenia by performing hematologic analyses of peripheral blood from control and cisplatin-treated mice with or without EA (**Figure 1A**). Cisplatin treatment induced leukopenia and EA prevented leukopenia and preserved the normal count of peripheral leukocytes. Next, we analyzed specific subpopulations of leukocytes as they are mainly composed of neutrophils, lymphocytes, and monocytes. Cisplatin decreased peripheral blood counts of all leukocytes but it was more detrimental to neutrophils and monocytes. We further confirmed our results with flow cytometry analyses of neutrophils (LY6G^+^), monocytes (LY6C^+^), and noted a similar effect on the subpopulations of lymphocytes T (CD3^+^) and B (CD19^+^) cells. EA was again effective at inhibiting cisplatin side effects and preserves normal peripheral counts of all these leukocytes and more protective on neutrophils and monocytes (**Figures 1B, C**). Cisplatin also induced about 25% mice body weight loss within 10 days, and EA preserved normal body weight over 14 days (**Figure 1D**). These results show that cisplatin induces leukopenia affecting all leukocytes although it was more detrimental to myeloid cells including neutrophils and monocytes, whereas EA preserved normal blood leukocyte counts.

**Figure 2:**
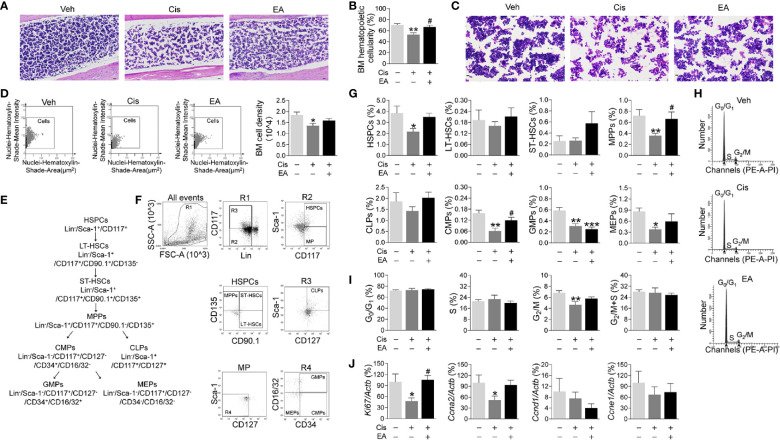
Electroacupuncture preserved hematopoiesis in mice with cisplatin chemotherapy. **(A)** Representative H&E staining of tibia BM from mice with control (Veh), cisplatin alone (Cis; 3 mg/kg), or with electroacupuncture (EA) treatment (scale bar=20.0 μm) and **(B)** Histogram representation of BM hematopoietic cellularity of H&E staining analyzed by Image-Pro Plus 6.0 software (*n=*4 per group). **(C)** Representative H&E staining of tibia BM from mice with Veh, cisplatin alone or with EA treatment at high configuration (scale bar=10.0 μm). **(D)** Representative HistoFAXS Tissue Analysis of BM cell nuclei hematoxylin-shade-mean intensity, and quantitative analysis of BM cell density (*n*=4 per group). **(E)** Flowchart of hematopoiesis and hematopoietic cells markers. **(F)** Representative flow cytometry analyses and **(G)** quantification of hematopoietic BM cell subpopulations (Positive cells events (%) = (the events in target gate/the total cell) × 100) (*n*=6 per group). **(H)** Representative PI nuclear staining flow cytometry analyses in BM cell cycle (G_0_/G_1_, S, G_2_/M phases) and **(I)** Quantification of PI nuclear staining of BM cells in G_0_/G_1_, S, G_2_/M phases by ModFit 3.1 software (*n*=6 per group). **(J)** Expression of cell cycle related genes in BM cells (*Ki67*: Veh, *n*=7; Cis, *n*=5; EA, *n*=7. *Ccna2*: *n*=7 per group. *Ccnd1*: Veh, *n*=5; Cis, *n*=4; EA, *n*=6. *Ccne1*: Veh, *n*=6; Cis, *n*=4; EA, *n*=5). Data are mean ± SEM, ^*^ *P <* 0.05, ^**^ *P <* 0.01, ^***^ *P <* 0.001 *vs* Veh; ^#^ *P <* 0.05 *vs* Cis.

Next, we analyzed the effects of cisplatin and EA in BM hematopoiesis. Histological hematoxylin & eosin (H&E) staining show normal BM morphology with proliferating hematopoietic cells in control mice. Cisplatin induced a sparse and scattered cell distribution, whereas EA preserved normal BM morphology (**Figure 2A**). We confirmed these results with semi-quantitative analyses BM hematopoietic cellularity showing that cisplatin decreased BM cells percentages, whereas EA improved it (**Figure 2B**). As shown in **Figures 2C, D**, we also performed the BM cell density at high configuration, and the results showed that cisplatin reduced BM cell counts, and EA treatment have increased tendency. Then, we analyzed the effects of cisplatin and EA in BM hematopoiesis by analyzing specific hematopoietic cell subpopulations (**Figure 2E**). Hematopoiesis starts with hematopoietic stem/*progenitor* cells (HSPCs; Lin^-^Sca-1^+^CD117^+^) undergoing a sequential differentiation into self-renewal *long-term* (LT-HSCs; Lin^-^/Sca-1^+^/CD117^+^/CD90.1^+^/CD135^-^), and *short-term* hematopoietic stem cells (ST-HSCs; Lin^-^/Sca-1^+^/CD117^+^/CD90.1^+^/CD135^+^), which differentiate into non-self-renewing *multipotent* progenitors (MPPs; Lin^-^/Sca-1^+^/CD117^+^/CD90.1^-^/CD135^+^). These progenitors can then differentiated into either common *lymphoid* (CLPs; Lin^-^/Sca-1^+^/CD117^+^/CD127^+^ for lymphocytes and NK cells) or common *myeloid* progenitors (CMPs; Lin^-^/Sca-1^-^/CD117^+^/CD127^-^/CD34^+^/CD16/32^-^), which ensuing differentiate into either *megakaryocytic/erythroid* (MEPs; Lin^-^/Sca-1^-^/CD117^+^/CD127^-^/CD34^-^/CD16/32^-^) or *granulocyte-macrophage* progenitors (GMPs; Lin^-^/Sca-1^-^/CD117^+^/CD127^-^/CD34^+^/CD16/32^+^ for neutrophils, monocytes, basophils, and eosinophils) (55, 56). Flow cytometry analyses showed that cisplatin was more detrimental in reducing HSPCs, MPPs, and myeloid ontogenesis (CMPs, GMPs, and MEPs), but not self-renewing stem cells (LT-HSCs, ST-HSCs) or lymphoid ontogenesis (CLPs). EA preserved the normal counts of all hematopoietic cells (MPPs, CMPs), and the proportion of HSPCs and MEPs have increased tendency, but not GMPs (**Figures 2F, G**). These results show that cisplatin inhibited BM hematopoiesis and specifically myeloid ontogenesis, whereas EA preserved BM hematopoiesis.

We next studied hematopoietic cell proliferation and cycle profile in BM by propidium iodide nuclear staining. Cisplatin inhibited hematopoietic cell proliferation by decreasing the transition from S to G_2_/M phase, whereas EA preserved normal cell proliferation (**Figures 2H, I**). At the molecular level, we analyzed the expression of cell cycle genes by quantitative RT-qPCR. Cisplatin specifically reduced the expression of *Ki67* and *Ccna2* without significantly affecting *Ccnd1* and *Ccne1*, whereas EA preserved normal expression of these genes (**Figures 2J**). These results show that cisplatin inhibits the progression of S into the G_2_/M phase by inhibiting DNA replication and the expression of critical factors such as *Ki67* (associated with ribosomal RNA synthesis) and *Ccna2 *(cyclin A2). Again, EA preserved normal cell proliferation of BM hematopoietic cells.

### Activated Bone Marrow Pathways in Cisplatin-Treated Mice by Electroacupuncture

**Figure 3:**
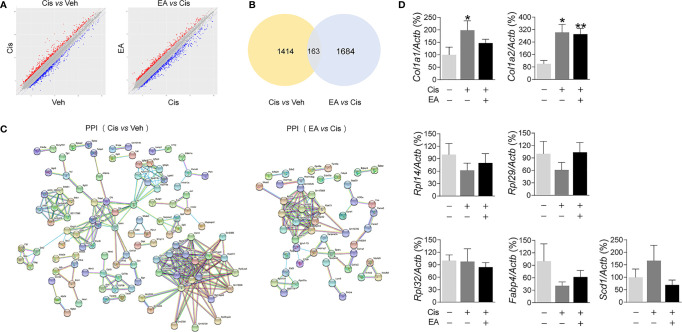
Analyses of expression and enrichment of electroacupuncture in the bone marrow of mice with cisplatin. **(A)** Scatter plots of differentially expressed genes (DEG)s in Cis *vs* Veh and EA *vs* Cis group Each probe is represented by a point with red and blue points showing up- and down-regulated genes defined above Log2 FC > 2. **(B)** Venn diagram and **(C)** PPI network analyses of DEGs results. **(D)** RT-qPCR analyses of factors related to extracellular matrix (*Col1a1*, *Col1a2*), ribosome (*Rpl14*, *Rpl29*, *Rpl32)*, and PPAR signaling (*Fabp4*, *Scd1)* (*Col1a1*, *Col1a2*: *n*=7 per group. *Rpl14*, *Rpl29*, *Scd1*: *n*=6 per group. *Rpl32*, *Fabp4*: Veh, *n*=6; Cis, *n*=5; EA, *n*=6). Data are mean ± SEM, ^*^ *P <* 0.05, ^**^ *P <*0.01 *vs* Veh.

We further analyzed the molecular mechanisms of cisplatin and EA by gene chip analyses (**Figure 3A**). Cisplatin modified the expression of 1,414 BM genes as compared to normal tissue, and EA modified 1,684 genes as compared to cisplatin (**Figure 3B**). Differential gene KEGG pathway analyses revealed that cisplatin main effects (*P* < 0.01; enrichment > 3) were activating pathways related to extracellular matrix receptor interaction, B cell and toll-like receptors signaling, the p53, PPAR signaling, osteoblast differentiation and NF-kB pathways (**Table 1**). KEGG analyses also showed the potential of EA to mainly activate pathways related to ribosome biogenesis (*P* < 0.01; enrichment > 28) (**Table 1**). KEGG analyses revealed 163 common differentially expressed genes (DEG) in both cisplatin and EA groups. The factors modulated by both cisplatin and EA further emphasizes the role of three major pathways (*P* < 0.01) related to ribosome biogenesis (*Rpl14*, *Gm6344*, *Rpl29*, *Rpl32*; enrichment > 12), PPAR signaling (*Fabp4*, *Scd1*; enrichment > 9), and collagen extracellular matrix receptor interaction (*Col1a1*, *Col1a2*, enrichment *> 8*) (**Table 1**). These results were consistent with the protein-protein interaction (PPI) analyses that revealed the potential of cisplatin to induce 71 genes mostly related to ribosome biogenesis (*Rps13*, *Rpl14*, *Rpl32*, *Rpl34*, > 15 counts) and collagen extracellular matrix (*Col1a1*, *Col1a2*, 3 counts/each) (**Figure 3C** and **Table 2**). EA was again protective against cisplatin and preserving the expression of 48 genes mostly related to ribosome biogenesis (*Rpl14*, *Rps11*, *Rps13*, *Rpl32*, *Rpl34*, > 10 counts) (**Table 2**). The levels of common DEG detected by gene chip in both cisplatin and EA further emphasized the role of ribosome biogenesis, collagen extracellular matrix receptor interaction, and PPAR signaling (**Additional File 2: Table S2**). These results were then confirmed by RT-qPCR. Cisplatin significantly induced *Col1a1, Col1a2*, expression as shown in KEGG analyses, whereas EA preserved *Col1a1* normal expression consistent with the gene chip analyses (**Figure 3D**). These results suggest that cisplatin induces type I collagen α1 chain (*Col1a1*) and disrupts BM extracellular matrix, whereas EA preserves normal collagen BM expression and extracellular matrix composition for normal hematopoiesis.

**Table 1:** KEGG enrichment of co-expressed DEGs.

Description	*P* value	Counts	Genes	Enrich factor
**KEGG enrichment of co-expressed DEGs in cisplatin *vs* control group**				
Extracellular matrix receptor interaction	<0.01	9	*Thbs1 Gp5 Reln Gp6 Col1a2 Col1a1 Gp9 Gp1ba Itgb3*	8.11
B cell receptor signaling pathway	<0.01	7	*Jun Cd79a Fos Blnk Cd79b Cd19 Cd72*	7.27
Hematopoietic cell lineage	<0.01	7	*Gp5 Gp9 Cd19 Gp1ba Il1a Itgb3 Il7r*	5.51
Toll-like receptor signaling pathway	<0.01	5	*Jun Cxcl9 Fos Ctsk Ifna4*	3.78
p53 signaling pathway	<0.01	4	*Thbs1 Ccng1 Cdkn1a Pten*	4.21
NF-kappa B signaling pathway	<0.01	5	*Cxcl12 Tnfrsf13c Blnk Lat Vcam1*	3.59
PPAR signaling pathway	<0.05	4	*Scd1 Fabp4 Lpl Adipoq*	3.52
Osteoclast differentiation	<0.05	6	*Jun Fos Ctsk Blnk Il1a Itgb3*	3.50
Th17 cell differentiation	<0.05	4	*Jun Fos Irf4 Lat*	2.93
Serotonergic synapse	0.07	4	*Gng11 Dusp1 Kcnj5 Alox12*	2.27
Apoptosis	0.08	4	*Jun Fos Ctsk Tuba4a*	2.20
Cellular senescence	0.09	5	*Mapkapk2 Slc25a5 Cdkn1a Il1a Pten*	2.01
**KEGG enrichment of co-expressed DEGs in EA *vs* cisplatin group**				
Ribosome biogenesis in eukaryotes	<0.01	33	*N-r5s100 Gm25212 N-r5s123 N-r5s134 Rn5s N-r5s128 N-r5s124 N-r5s136 N-r5s121 Gm23284 N-r5s117 Gm22109 N-r5s108 N-r5s122 N-r5s105 Gm22291 Rmrp N-r5s143 N-r5s139 N-r5s111 N-r5s103 N-r5s138 Gm25018 N-r5s146 N-r5s113 N-r5s142 N-r5s149 Gm26391 N-s5s110 N-r5s144 N-r5s133 N-r5s104 N-r5s141*	28.44
Biosynthesis of unsaturated fatty acids	0.09	1	*Scd1*	3.12
Extracellular matrix receptor interaction	0.11	2	*Col1a1 Col1a2*	2.41
PPAR signaling pathway	0.11	2	*Fabp4 Scd1*	2.35
Retrograde endocannabinoid signaling	0.14	3	*Ndufb9 Nd2 Ndufa9*	2.00
**KEGG enrichment of co-expressed DEGs in cisplatin *vs* control group and EA *vs* cisplatin group**				
Ribosome	<0.01	4	*Rpl14 Gm6344 Rpl29 Rpl32*	12.20
PPAR signaling pathway	<0.01	2	*Scd1 Gabp4*	9.19
Extracellular matrix receptor interaction	<0.01	2	*Col1a1 Col1a2*	8.65

Enrich factor = (the number of DEGs in a term/the total number of DEGs) / (the total gene number in a term of database/the total number of genes in the database).

**Table 2:** The node counts between proteins with PPI.

Nodes	Counts	Nodes	Counts	Nodes	Counts
**The node counts between proteins with PPI in cisplatin *vs* control group**					
*Rps13*	21	*Cfd*	7	*Cd79a*	4
*Rpl14*	19	*Gng11*	7	*Cd79b*	4
*Rpl32*	19	*Igfbp4*	7	*Dcn*	4
*Rpl34*	16	*Igfbp5*	7	*Serpine2*	4
*F5*	14	*Igfbp7*	7	*Blnk*	3
*Rpl13*	13	*Lgals1*	7	*Brix1*	3
*Rpl27-ps3*	13	*Thbs1*	7	*Col1a1*	3
*Rps27rt*	13	*Bc117090*	6	*Col1a2*	3
*Etf1*	12	*Ccl9*	6	*Ctsg*	3
*Rpl36*	12	*Clu*	6	*Fos*	3
*Rpl29*	11	*Gm5416*	6	*Ftl1*	3
*Pf4*	10	*Gm5483*	6	*Gm10709*	3
*Ppbp*	10	*mCG_130165*	6	*Gm5786*	3
*Rpl10*	10	*Rpl9-ps6*	6	*Gp1ba*	3
*Gm10269*	9	*Stfa1*	6	*Gp5*	3
*Gm17669*	9	*Stfa3*	6	*Gp9*	3
*Sparc*	9	*Cd19*	5	*H2afv*	3
*Gm10036*	8	*Cxcl12*	5	*Mpo*	3
*Rpl13-ps3*	8	*Cxcl9*	5	*Psma5*	3
*Apol10a*	7	*Gm9396*	5	*Rpl36-ps3*	3
*Apol11a*	7	*Stfa2l1*	5	*Vcl*	3
*Apol11b*	7	*Cct2*	4		
**The node counts between proteins with PPI in EA *vs* cisplatin group**					
*Rpl14*	11	*Gm17669*	6	*Col1a1*	2
*Rps11*	11	*Cst3*	4	*Col1a2*	2
*Rps13*	11	*Serping1*	4	*Ighv1-73*	2
*Rpl32*	11	*Sparc*	4	*Lsm5*	2
*Rpl34*	10	*Apol10a*	3	*mt-Nd2*	2
*Rps26-ps1*	9	*Apol11a*	3	*Ndufa9*	2
*Rpl29*	8	*Apol11b*	3	*Psmb7*	2
*Gm10020*	7	*Gm10709*	3	*Serpina3n*	2
*Gm10126*	7	*H3f3a*	3		
*Rpl10*	7	*C1qb*	2		

### Sympathetic Nerve Released PACAP Mediating Electroacupuncture Alleviation of Cisplatin-Induced Leukopenia

**Figure 4:**
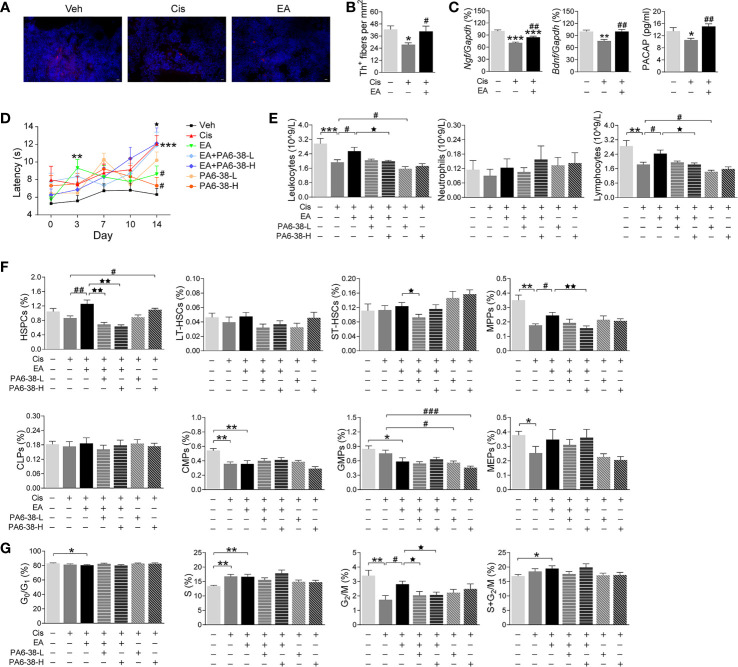
Neurogenic PACAP mediated electroacupuncture-induced protection to cisplatin. **(A)** Representative immunofluorescence images (Scale bar=20.0 μm) and **(B)** Quantification of sympathetic Th^+^ fibers (red) and nuclear (blue) in the BM of the experimental mice (*n=*4 per group). **(C)** Expression analyses of neurotrophic factors (*Ngf*, *Bndf*: Veh, *n*=7; Cis, *n*=6; EA, *n*=7. PACAP: Veh, *n*=5; Cis, *n*=6; EA, *n*=6). **(D)** Representation of the latency time (seconds) in hot-plate tests of mice treated with control (Veh), cisplatin (Cis; 3mg/kg), and cisplatin + electroacupuncture (EA) without or with PACAP6-38 (a blocker for PACAP receptor, PAC1) at low (10 μg/kg) or high (100 μg/kg) concentrations (Cis, *n*=7; other groups, *n*=8), *P* values were calculated using two-way repeated-measures ANOVA. **(E)** Peripheral blood counts of specific subpopulation of leukocytes (Veh, Cis, EA: *n*=6; other groups, *n*=7). **(F)** Analyses of hematopoietic BM subpopulation cells (Veh, Cis, EA, EA+PA6-38-L, EA+PA6-38-H: *n*=7; PA6-38-L, *n*=8, PA6-38-H, *n*=6). **(G)** Quantification of PI nuclear staining of BM cells (Veh, *n*=8; Cis, *n*=7; EA, *n*=8; EA+PA6-38-L, *n*=8; EA+PA6-38-H, *n*=7; PA6-38-L, *n*=8; PA6-38-H, *n*=7). Data are mean ± SEM ^*^ *P <* 0.05, ^**^ *P <* 0.01, ^***^ *P <* 0.001 *vs* Veh; ^#^ *P <* 0.05, ^##^ *P <* 0.01, ^###^ *P <* 0.001 *vs* Cis; ^⋆^ *P <* 0.05, ^⋆⋆^ *P <* 0.01 *vs* EA.

We next reasoned that cisplatin-induced neurotoxicity may affect hematopoiesis, and EA may preserve BM sympathetic neuromodulation. Thus, we analyzed the sympathetic fibers in BM sections by staining tyrosine hydroxylase (Th), the enzyme that converts tyrosine to dopamine essential for catecholamine biosynthesis in sympathetic innervations. These results showed the significant neurotoxicity induced by cisplatin, and the potential of EA to preserve BM sympathetic innervations (**Figures 4A, B**). Then, we performed RT-qPCR analyses to determine the neurogenic factors mediating EA-induced neuroprotection. Protein expression was confirmed by ELISA analyses. Cisplatin inhibited the production of critical neurogenic factors but especially nerve growth factor (*Ngf*), brain-derived neurotrophic factor (*Bdnf*), and PACAP. EA preserved normal production of all these factors, but was more effective in inducing PACAP expression (**Figure 4C**). Thus, we reasoned that PACAP may contribute to EA-induced neuroprotection during chemotherapy, and we analyzed whether PACAP inhibition prevents EA-induced neuroprotection using functional analyses of nociception. Previous studies reported that cisplatin neurotoxicity induces peripheral nerve injury affecting nociception (54). Thus, we analyzed whether EA preserves sensory nerve activity using thermal pain tests, and whether this effect is mediated by PACAP. Cisplatin increased mice latency time in the hot-plate tests showing neurotoxicity preventing thermal pain, whereas EA preserved thermal nociception (**Figure 4D**). Next, we analyzed whether PACAP is required for EA-induced neuroprotection by inhibiting the specific receptor for PACAP, PAC1. PACAP6-38, a competitive PAC1 inhibitor, abrogated the potential of EA to preserve nociception in thermal tests in a concentration-dependent manner (**Figure 4D**). Then, we analyzed whether the effects of PAC1 on neuroprotection correlated with hematopoiesis. Similar to neuroprotection, EA prevented cisplatin-induced leukopenia, but not in mice pretreated with high doses of PAC1 inhibitor (**Figure 4E**). Likewise, PAC1 inhibitor also prevented the potential of EA to preserve BM hematopoiesis and counts of HSPCs and myeloid progenitors (MPPs) during cisplatin chemotherapy (**Figure 4F**). Control treatments with PACAP6-38 itself affected neither BM hematopoiesis nor HSPCs/MPPs counts. Furthermore, PAC1 inhibitor also prevented the potential of EA to preserve hematopoietic cell proliferation (**Figure 4G**). Together, these results show that inhibition of PACAP receptor PAC1 prevents the protective effects of EA during cisplatin chemotherapy, suggesting that the protective effects of EA are mediated by PACAP production.

**Figure 5:**
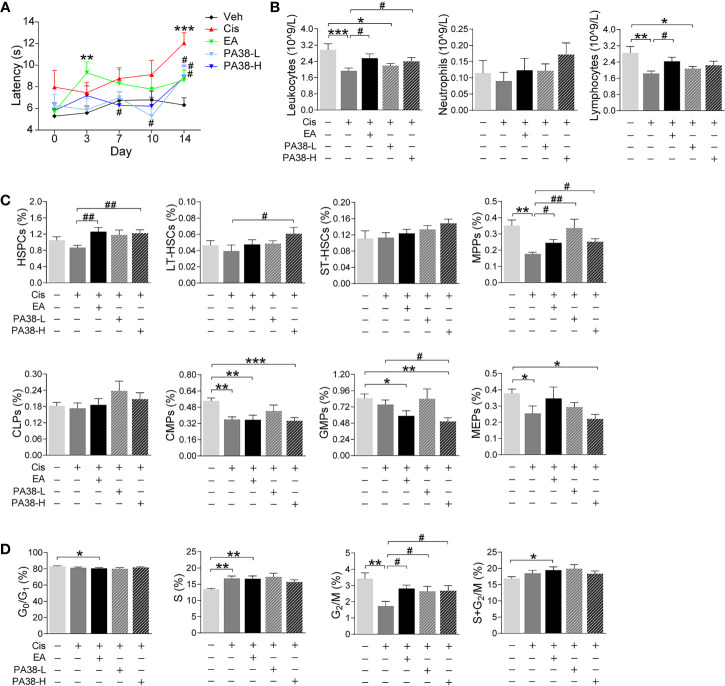
PAC1-agonist mimics electroacupuncture-induced protection to cisplatin. **(A)** Representation of the latency time (seconds) in hot-plate tests of mice with control (Veh), cisplatin (Cis; 3 mg/kg), EA (cisplatin + electroacupuncture), cisplatin mice were treated with low (10 μg/kg) or high (50 μg/kg) concentrations PAC1-agonist, PACAP1-38 (Veh, *n*=8; Cis, *n*=7; EA, *n*=8; PA38-L, *n*=8; PA38-H, *n*=7), *P* values were calculated using two-way repeated-measures ANOVA. **(B)** Peripheral blood counts of specific subpopulation of leukocytes ((Veh, Cis, EA: *n*=6; other groups, *n*=7). **(C)** Analyses of hematopoietic BM cell subpopulation (Veh, Cis, EA: *n*=7; PA38-L, PA38-H: *n*=8). **(D)** Quantification of PI nuclear staining of BM cells (Veh, *n*=8; Cis, *n*=7; EA, *n*=8; PA38-L, *n*=8; PA38-H, *n*=7). Data are mean ± SEM, ^*^ *P <* 0.05, ^**^ *P <* 0.01, ^***^ *P <* 0.001 *vs* Veh; ^#^ *P <* 0.05, ^##^ *P <* 0.01 *vs* Cis.

Next, we reasoned that PAC1-agonists may mimic the protective effects of EA during cisplatin chemotherapy. PAC1-agonist, PACAP1-38, mimics EA-induced neuroprotection and preserves thermal nociception in cisplatin-treated mice in a concentration-dependent manner (**Figure 5A**). The high dose of PACAP1-38 preserves BM hematopoiesis and normal peripheral counts of leukocytes, including neutrophils and lymphocytes (**Figure 5B**). The high dose of PAC1-agonist also mimics the potential of EA to preserve hematopoiesis including HSPCs and myeloid progenitors (MPPs) but not GMPs (**Figure 5C**). The high and low dose of PAC1-agonist also preserved BM hematopoietic cell proliferation through the G_2_/M phase (**Figure 5D**). Thus, treatment with high dose of PAC1-agonist, PACAP1-38, mimicked the potential of EA to preserve thermal nociception, peripheral counts of leukocytes, BM myeloid ontogenesis, and hematopoietic cell proliferation in mice with cisplatin chemotherapy.

### Preservation of BM Hematopoiesis in Lung Carcinoma Mice by Electroacupuncture

**Figure 6:**
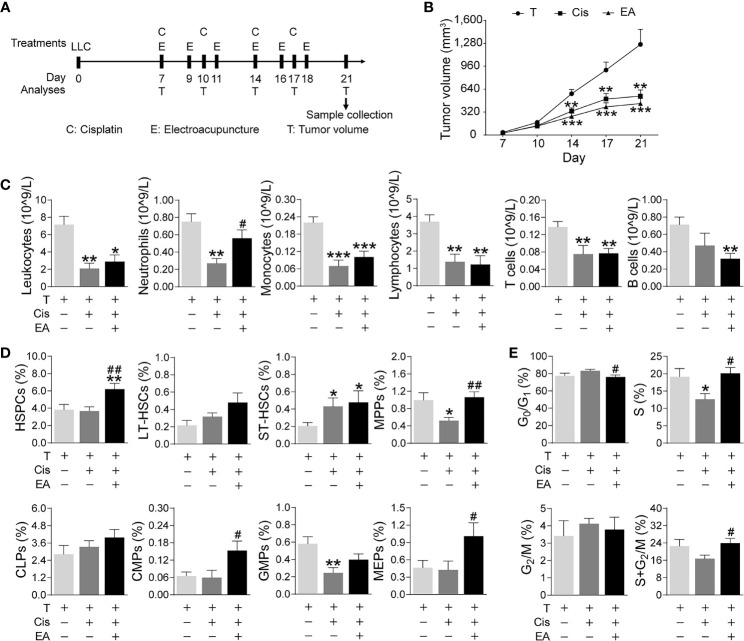
Electroacupuncture restores hematopoiesis in cancer mice during cisplatin chemotherapy. **(A)** Experimental flowchart depicting the time of treatments of tumor (LLC) cells at day 0, cisplatin (C), electroacupuncture (E), and analyses of tumor volume (T) and sample collection. **(B)** Tumor growth curve (*n*=9 per group), *P* values were calculated using two-way repeated-measures ANOVA. **(C)** Peripheral blood counts of specific subpopulation of leukocytes (leukocytes, lymphocytes: T, *n*=8; Cis, *n*=6; EA, *n*=8. neutrophils, monocytes, T and B lymphocytes: T, *n*=8; Cis, *n*=6; EA, *n*=7). **(D)** Analyses of hematopoietic BM cell subpopulation (*n* =9 per group). **(E)** Quantification of PI nuclear staining of BM cells (*n*=9 per group). Data are mean ± SEM, ^*^ *P <* 0.05, ^**^ *P <* 0.01, ^***^ *P <* 0.001 *vs* Veh; ^#^ *P <* 0.05, ^##^ *P <* 0.01 *vs* Cis.

We next analyzed the effects of EA in cancer mice with LLC cells. Mice were injected LLC cells, cisplatin chemotherapy with or without EA was started one week later, and tumor growth and hematopoiesis were analyzed at different time points (**Figure 6A**). Tumor volume dramatically increases after 14 days, and cisplatin treatment (5 mg/Kg; i.p.) significantly reduces tumor growth by over 60% by day 21 (**Figure 6B**). EA did not prevent the potential of cisplatin to inhibit tumor growth, actually EA showed a tendency to further decrease tumor growth to some extent as compared to cisplatin treatment alone. Cisplatin also induces peripheral leukopenia inhibiting all leukocyte subpopulations including neutrophils, monocytes, and lymphocytes, and it was more detrimental on T (CD3^+^) than B (CD19^+^) lymphocytes in cancer mice. Furthermore, EA diminished leukopenia and neutropenia but not monocytopenia and lymphopenia in cancer mice (**Figure 6C**). Cisplatin inhibited hematopoiesis at different levels and significantly reduced the counts of multipotent (MPPs) and GMPs in cancer mice. EA preserved normal levels of both MPPs and GMPs in cancer mice. Furthermore, EA increased the levels of HSPCs, myeloid (CMPs), and megakaryocytic/erythroid progenitors (MEPs) in cancer mice (**Figure 6D**). At the cellular level, cisplatin significantly decreased BM cell counts in S phase, whereas EA preserved normal cell proliferation through the cell cycle in cancer mice (**Figure 6E**). These results show that EA diminished cisplatin-induced leukopenia and preserves BM hematopoiesis in cancer mice with Lewis lung carcinoma cells.

## Discussion

Despite the profuse clinical evidence showing the potential of EA to relieve leukopenia during chemotherapy, its mechanism is unknown, and thus why it is effective in some patients but not in others with similar symptoms and how the treatment can be improved. EA activates mechanisms that have physiologic limitations, and they are ineffective in patients with multiple comorbidities (57, 58). One typical example is that EA on ST36 improves organ function and survival in experimental sepsis by inducing dopamine production in the adrenal glands (49, 59). However, many septic patients have adrenal insufficiency, and thus they render insufficient dopamine production for EA to induce significant effects (49, 60, 61). Chemotherapy is another major clinical challenge that causes neurotoxicity, anemia, and immunosuppression that limit anti-tumor efficacy. Here, we show that EA on ST36 and SP6 prevents neurotoxicity, preserves BM hematopoiesis, and myeloid ontogenesis during cisplatin chemotherapy. EA induces neuro and immune protection by inducing neurogenic production of PACAP, which preserves BM hematopoiesis *via* PAC1 receptor. Thus, PAC1-agonists mimic EA potential to preserve BM hematopoiesis during chemotherapy and may provide therapeutic advantages to treat cancer patients with advanced neurotoxicity and neuropathies limiting EA efficacy.

Cisplatin is an effective chemotherapy treatment toxic to proliferating cells such as cancer cells. However, cisplatin is not specific for cancer cells and it also inhibits BM hematopoietic cells inducing anemia and immunosuppression that prevent anti-tumor immune responses (58, 62–64). Low concentrations of cisplatin (3 mg/kg) in normal mice decreased blood counts of all leukocytes but specially neutrophils and monocytes. Higher concentrations of cisplatin (5 mg/kg) are required to induce similar effects in cancer mice probably because it is absorbed by the cancer cells. In cancer mice, cisplatin also inhibited all leukocytes subpopulations and it was more detrimental to T than B lymphocytes. These results further reveal the potential of cisplatin to induce immunosuppression and limit anti-tumor immune responses.

Cisplatin causes leukopenia by inhibiting hematopoiesis. Cisplatin inhibited hematopoietic stem/progenitor, multipotent progenitors, and myeloid ontogenesis (CMPs, GMPs, and MEPs), but not self-renewing stem cells (LT-HSCs, ST-HSCs) or lymphoid ontogenesis (CLPs) in normal mice. In cancer mice, cisplatin induced similar results and inhibited multipotent progenitors and myeloid ontogenesis of GMPs, and thus validate our models to recapitulate leukopenia as shown in cancer patients (62). However, cisplatin did not inhibit megakaryocytic/erythroid progenitors in cancer mice because the lung carcinoma cells already prevent MEPs as compared to normal mice. These results concur with the peripheral blood counts as cisplatin inhibits myeloid ontogenesis and therefore neutrophils and monocytes in normal and cancer mice. Cisplatin inhibits hematopoiesis by binding to the nuclear DNA of proliferative hematopoietic cells and inducing an in-chain DNA cross-linking that forms a ternary complex of DNA-platinated oligonucleotide-HMGB1 (high-mobility group Box one protein) that blocks DNA replication and cell proliferation (65, 66). Thus, cisplatin prevents the transition S to G_2_/Mitosis phase as shown in normal mice, whereas higher concentrations in cancer mice were more effective at early stages and decrease cell counts in the S phase. This effect is also due to the potential of cisplatin to inhibit the expression of critical proteins related to the cell cycle. Our results show that cisplatin inhibited *Ccna2* expression of Cyclin A2, which is normally expressed in dividing somatic cells to control the G_1_ to S transition as shown in our results with cancer mice. These results reveal the detrimental side effects of cisplatin in hematopoiesis during chemotherapy and the clinical need to develop safe complementary treatments to prevent immunosuppression in cancer patients.

Multiple clinical studies have confirmed the potential of acupuncture to treat anemia and leukopenia during chemotherapy (17, 20), but the use and efficacy of EA are still moot because of the weak response in many patients. The mechanism of EA is still unknown and thus why it is effective in many patients but not in others with similar symptoms. According to traditional Chinese medicine, acupuncture at ST36 and SP6 have the effect of tonifying blood. Several studies show that stimulation of these two acupoints protects against chemotherapy induced anemia, leukopenia, and other peripheral neuropathies (67–71). Our results show that EA ST36 and SP6 inhibited the most detrimental effects of cisplatin in normal and cancer mice. EA preserved normal peripheral counts of all leukocytes, and BM counts all hematopoietic cells (HSPCs, MPPs, CMPs, and MEPs) but not GMPs in normal mice. In cancer mice, EA halted leukopenia and neutropenia and preserved normal counts of multipotent (MPPs) and GMPs. Actually, EA not only prevented the effects of cisplatin but also some of the effects of cancer on hematopoiesis. As LLC cells decreased BM counts of common myeloid and megakaryocytic/erythroid progenitors in cancer mice, EA restored normal counts of BM hematopoietic cells even if the treatment was started a week after the cancer onset.

Regarding the molecular mechanism of EA, gene chip results suggested that EA may modulate the BM extracellular matrix (ECM) and ribosome signaling pathway (cisplatin vs control, EA *vs* cisplatin, EA *vs* control). EA restored BM hematopoiesis despite the effects of cancer and chemotherapy by regulating type I collagen α1 chain (*Col1a1*). Actually, *Col1a1* is often increased in cancer patients and disrupts BM hematopoiesis and favors immunosuppression and tumor progression (72–74). Thus, the potential of EA to halt *Col1a1* and abnormal collagen production can explain its potential to restore hematopoiesis and ameliorate the cancer inhibition of common myeloid and megakaryocytic/erythroid progenitors as discussed above in cancer mice. These results may suggest that EA can be more effective than anticipated for cancer treatment and not only beneficial to patients with chemotherapy.

Furthermore, EA restores hematopoiesis by preserving normal hematopoietic cell proliferation and production critical factors regulating the cell cycle such as *Ccna2* expression of Cyclin A2. One significant advantage of EA is its potential to activate specific neuronal networks and induce local effects. Thus, EA preserved *Ccna2* expression and hematopoietic cell proliferation in the BM without enhancing tumor proliferation (75). In addition to *Ccna2*, EA also preserved the normal expression of *Ki67* for ribosomal RNA synthesis. These results concur with the KEGG and protein-protein interaction analyses showing the potential of EA to preserve multiple factors associated with ribosomal RNA synthesis. Ribosomes are critical intracellular translational machinery responsible for protein synthesis and cellular proliferation. Eukaryotic 80S ribosomes are composed of two subunits, a 40S decoding subunit, and a large 60S subunit that catalyzes the peptide bonds (76). Chemotherapy drugs inhibit ribosomes at different levels, whereas oxaliplatin induces DNA damage with nucleolar and ribosomal disruption as shown by proteomic profiling (77), cisplatin modifies ribosomal mRNA *via* 1xr1-TOR signaling pathway to prevent protein synthesis. Ixr1 is an HMGB protein that regulates the hypoxic regulon and controls the oxidative stress response or re-adaptation of catabolic and anabolic fluxes in hypoxia. Ixr1 binds with high affinity to cisplatin-DNA adducts and, thus, cisplatin treatment mimics IXR1 deletion, and prevents ribosome biogenesis. Ixr1 is critical to regulating multiple transcriptional factors that respond to nutrient availability and stress stimuli through the TOR and PKA pathways (78, 79). Our analyses showed cisplatin inhibiting multiple factors affecting both 40S and 60S ribosome subunits, whereas EA preserved their normal expression.

Our molecular analyses also show the potential of EA to modulate over 1,600 BM genes that are mainly related to extracellular matrix receptor interaction, B cell and toll-like receptors signaling, and the p53 and NF-kB pathways. Indeed, the extracellular matrix is critical to hematopoiesis and the response of hematopoietic cells to neurotransmitters and growth factors (24, 80). For instance, fibronectin is important for the adhesion and proliferation of hematopoietic and erythroid progenitors (81), whereas adiponectin can inhibit myelomonocytic cell expansion (82) and *Col1a1* and *Col1a2* are produced by BM stromal cells to define BM hematopoietic niche microenvironment (83, 84). Our RT-qPCR analyses showed that cisplatin activates *Col1a1 and Col1a2*, and EA preserved normal *Col1a1* production. The potential of EA to modulate *Col1a1* may be more significant than anticipate and not only beneficial to patients with chemotherapy. The control of *Col1a1* by EA can explain its potential to restore hematopoiesis and ameliorate the cancer inhibition of common myeloid and megakaryocytic/erythroid progenitors as discussed above in cancer mice. Our results warrant future studies to determine the role of this mechanism in hematopoietic cell translocation and egress and their clinical implications in cancer progression.

The main effects of EA are mediated by the nervous system, which is critical to coordinate BM hematopoiesis for physiological homeostasis. Many studies have shown that chemotherapy drugs such as cisplatin are neurotoxic and damage BM autonomic nerves compromising hematopoiesis (45). Thus, ablation of sensory nerves with capsaicin also reduces BM cellularity and causes leukopenia (85). Our results show that cisplatin induced neurotoxicity and inhibited the production of multiple neurogenic factors such as *Ngf*, *Bdnf*, and PACAP, whereas EA induced sympathetic neuroprotection and preserved the production of these factors. Of note, previous studies reported that the 28-38 tail of PACAP is important for blood transportation, BBB crossing, and degradation by plasma endopeptidases (31, 33). Furthermore, PACAP has two isoforms, PACAP27 and PACAP38, with the latter being the dominant in mammalian tissue at normal physiological conditions. However, their respective levels change in different physiological and pathological conditions. For instance, PACAP27 and PACAP38 levels were lower in lung cancer samples than in healthy tissue. Likewise, our present study shows lower PACAP levels during cisplatin chemotherapy. Given that our PACAP ELISA kit recognizes both PACAP27 and PACAP38, future detailed studies will be required to determine the differential role of PACAP27 and PACAP38 in chemotherapy, neuromodulation of bone marrow hematopoiesis, and electroacupuncture.

Our previous studies showed that PACAP-specific receptor (PAC1) is strongly expressed on HSPCs of murine BM, and adcyap1^−/−^ mice exhibited lower MPP populations and cell frequency in the S-phase of the cell cycle. Exogenous PACAP38 increased the numbers of colony forming unit-granulocyte/macrophage progenitor cells (CFU-GM) derived from HPSCs, and increased Cyclin D1 and Ki67 expression, and these effects were prevented by the PAC1 antagonist. Of note, the direct sympathetic regulation of HSPCs proliferation is also evidence by the fact that PACAP is not produced by BM cells, but secreted from the sympathetic terminals (42). In this study, our results showed PACAP is a critical neurogenic factor mediating the protective effects of EA during chemotherapy. We showed that EA-induced PACAP expression in BM is critical to sympathetic nerve neuroprotection during cisplatin chemotherapy, and neurogenic PACAP derived from BM sympathetic nerve terminals mediated the protective effects of EA in cisplatin chemotherapy.

Inhibition of PACAP receptor PAC1 with high dose of PACAP6-38, abrogated the potential of EA to preserve thermal nociception, BM hematopoiesis, hematopoietic cell proliferation, and peripheral leukopenia. Conversely, pharmacologic activation of PAC1-agonist, with high dose of PACAP1-38 mimics EA-induced neuroprotection and preserved thermal nociception in cisplatin-treated mice. PACAP1-38 also preserved BM hematopoiesis, hematopoietic cell proliferation, and peripheral leukocyte levels. Furthermore, PACAP6-38 treatment can decrease the hematopoiesis in cisplatin-treated mice, probably by blocking the hematopoiesis promoting effect of the remaining PACAP secreted from injured sympathetic nerve terminal in BM. As shown in previous studies, activated PAC1 can interact with Gαs stimulating adenylyl cyclase leading to elevated cAMP, protein kinase A activation to promote neuronal survival in cerebellar granule neurons (86). Meanwhile, PAC1 signaling also stimulated the proliferation of adult mouse neural progenitor cells through PKC-dependent pathway (31, 87). The potential pathway maybe involve that activated PAC1 can interact with Gαq stimulating PLC causing phosphatidyl inositol turnover. The diacylglycerol activates protein kinase C leading to Src phosphorylation to activate matrix metalloprotease metabolizing transforming growth factor-α (TGF-α) from inactive precursors, leading to the tyrosine phosphorylation of the epidermal growth factor receptor to activate Ras and Raf, resulting in the tyrosine phosphorylation of mitogen/extracellular signal-regulated kinase and extracellular signal-regulated kinase to increase cellular proliferation (31, 88). The effects and molecular mechanism of PAC1 receptor in mediating preservation of BM hematopoiesis in lung carcinoma mice by EA needs further investigation. In conclusion, our results indicate that PAC1 signaling may be one of the mechanisms induced by EA to protect against cisplatin-induced neurotoxicity and immunosuppression in cancer patients, and PAC1-agonists may provide therapeutic advantages to treat patients with advanced neurotoxicity or neuropathies limiting EA efficacy.

## Data Availability Statement

The datasets presented in this study can be found in online repositories. The names of the repository/repositories and accession number(s) can be found at: NCBI BioProject, accession no: PRJNA687726.

## Ethics Statement

The animal study was reviewed and approved by Animal Care and Use Committee of Tianjin University of Traditional Chinese Medicine.

## Author Contributions

ZX and LU conceived the project. SLi, JH, JW, SLu, BW, YGong, SQ, SW, YF and SZ performed the experiments. YL, SLi, YMG and JH performed the data analysis. ZX and YGuo provided administrative, technical, or material support. SLi and JH wrote the initial manuscript draft. ZX and LU analyzed data, organized data presentation, and completed manuscript writing and preparation. All authors contributed to the article and approved the submitted version.

## Funding

This work was financially supported by the National Natural Science Foundation of China (grant numbers 81704146, 82030125, 82074534). Research Project of Tianjin Municipal Health Commission on Traditional Chinese Medicine and Integrative Medicine (grant number 2019140), Graduate Research Innovation Project of Tianjin University of Traditional Chinese Medicine (grant numbers YJSKC-20201009, YJSKC-20201029), and LU are supported by the NIH R01-GM114180.

## Conflict of Interest

The authors declare that the research was conducted in the absence of any commercial or financial relationships that could be construed as a potential conflict of interest.

## Publisher’s Note

All claims expressed in this article are solely those of the authors and do not necessarily represent those of their affiliated organizations, or those of the publisher, the editors and the reviewers. Any product that may be evaluated in this article, or claim that may be made by its manufacturer, is not guaranteed or endorsed by the publisher.

## Abbreviations

ANOVA, Analysis of variance; BM, bone marrow; Bdnf, brain derivedneurotrophic factor; CLPs, common lymphoid progenitors; CSF, colonystimulating factor; CMPs, common myeloid progenitors; DEG, common differentially expressed genes; EA, electroacupuncture; FBS, fetal bovine serum; FC, fold change; GMPs, granulocyte-macrophage progenitors; HSCs, hematopoietic stemcells; H&E, Hematoxylin & Eosin; HSPCs, hematopoietic stem/progenitor cells; HMGB1, high-mobility group Box 1; i.p, intraperitoneal; KEGG, Kyoto Encyclopedia of Genes and Genomes; LLC, lewis lung carcinoma; LT-HSCs, longterm HSCs; MPPs, multipotent progenitors; MEPs, megakaryocytic/erythroid progenitors; Ngf, nerve growth factor; PACAP, pituitary adenylate cyclase activating polypeptide; PBS, phosphate-buffered saline; PI, propidium iodide; PPI, protein-protein interaction; RT-qPCR, reverse transcription-quantitative and polymerase chain reaction; RIN, RNA integrity number; SEM, standard error of mean; ST-HSCs, short term HSCs; Th, tyrosine hydroxylase; TGF-a, transforming growth factor-a; COL1A1, type I collagen a1 chain; VIP, vasoactive intestinal peptide.
