# Supplementary material for: PAC1 Receptor Mediates Electroacupuncture-Induced Neuro and Immune Protection During Cisplatin Chemotherapy
Source: Front Immunol. 2021 Sep 6;12:714244. doi: 10.3389/fimmu.2021.714244 (PMC8450570; doi:10.3389/fimmu.2021.714244)
Supplement: Supplementary file 1 [file Table_1.docx]

Supplementary Material

# Supplementary Tables

**Table 1. The gene-specific primers used in this study.**

| Gene | Forward primer (5′→3′) | Reverse primer (5′→3′) | Product size |
| --- | --- | --- | --- |
| *Ki67* | CATGCAAACTCTCCCTGTACCA | TCGCTGAATTGGAAAGTACGGA | 217 |
| *Ccna2* | CCCAGTACTTCCTGCACCTG | GTTGTGCCAATGACTCAGGC | 189 |
| *Ccnd1* | TGCGTGCAGAAGGAGATTGT | CTTCTTCAAGGGCTCCAGGG | 150 |
| *Ccne1* | TATGGTGTCCTCGCTGCTTC | GGGTCTGGATGTTGTGGGAG | 202 |
| *Col1a1* | CTCAAGATGTGCCACTCTGACT | ACCTGTCTCCATGTTGCAGTAG | 105 |
| *Col1a2* | TCAGAACATCACCTACCACTGC | AGCCATCGACTAGGACAGAGTA | 155 |
| *Rpl14* | AGAACAGGGCTTTAGTGGATGG | CTGTGTGGGAACTTGAGGATGA | 103 |
| *Rpl29* | GAAATGGCATCAAGAAACCCCG | TGCATCTTCTTCAGGCCTTTCT | 121 |
| *Rpl32* | AGTTCCTGGTCCACAATGTCAA | GTGCTGCTCTTTCTACAATGGC | 123 |
| *Fabp4* | CAGCGTAAATGGGGATTTGGTC | TGATGCTCTTCACCTTCCTGTC | 125 |
| *Scd1* | TTCACCACGTTCTTCATCGACT | ACTCCCGTCTCCAGTTCTCTTA | 111 |
| *Ngf* | GATCTAGACTTCCAGGCCCATG | ACTGTCACACACTGAGAACTCC | 111 |
| *Bdnf* | GGCCCAACGAAGAAAACCAT | GTTTGCGGCATCCAGGTAAT | 131 |
| *Actb* | CATCCGTAAAGACCTCTATGCCAAC | ATGGAGCCACCGATCCACA | 171 |
| *Gapdh* | TGTGTCCGTCGGATCTGA | TTGCTGTTGAAGTTCGCAGGAG | 150 |

**Table 2. The common DEG levels detected by gene chip in cisplatin *vs* control group and EA *vs* cisplatin group.**

| Genes | Normalized signal (log2) (Veh) | Normalized signal (log2) (Cis) | Normalized signal (log2) (EA) |
| --- | --- | --- | --- |
| *Rpl14* | 14.86 | 16.07 | 17.18 |
| *Rpl29* | 13.01 | 14.2 | 15.27 |
| *Rpl32* | 16.58 | 17.74 | 19.04 |
| *Col1a1* | 6.87 | 9.15 | 7.25 |
| *Col1a2* | 6.26 | 8.32 | 6.74 |
| *Fabp4* | 7.74 | 9.76 | 8.66 |
| *Scd1* | 8.31 | 10.05 | 8.96 |
